# Advances in preclinical hematopoietic stem cell models and possible implications for improving therapeutic transplantation

**DOI:** 10.1002/sctm.20-0294

**Published:** 2020-10-15

**Authors:** Ellen Fraint, Bianca A. Ulloa, María Feliz Norberto, Kathryn S. Potts, Teresa V. Bowman

**Affiliations:** ^1^ Department of Pediatrics Children's Hospital at Montefiore Bronx New York USA; ^2^ Department of Developmental and Molecular Biology Albert Einstein College of Medicine Bronx New York USA; ^3^ Gottesman Institute for Stem Cell Biology and Regenerative Medicine Albert Einstein College of Medicine Bronx New York USA; ^4^ Department of Medicine (Oncology) Albert Einstein College of Medicine and Montefiore Medical Center Bronx New York USA

**Keywords:** graft‐vs‐host disease, hematopoietic stem cells, lineage tracing, preclinical models, transplantation

## Abstract

Hematopoietic stem cell transplantation (HSCT) is a treatment for many malignant, congenital, and acquired hematologic diseases. Some outstanding challenges in the HSCT field include the paucity of immunologically‐matched donors, our inability to effectively expand hematopoeitic stem cells (HSCs) ex vivo, and the high infection risk during engraftment. Scientists are striving to develop protocols to generate, expand, and maintain HSCs ex vivo, however these are not yet ready for clinical application. Given these problems, advancing our understanding of HSC specification, regulation, and differentiation in preclinical models is essential to improve the therapeutic utility of HSCT. In this review, we link biomedical researchers and transplantation clinicians by discussing the potential therapeutic implications of recent fundamental HSC research in model organisms. We consider deficiencies in current HSCT practice, such as problems achieving adequate cell dose for successful and rapid engraftment, immense inflammatory cascade activation after myeloablation, and graft‐vs‐host disease. Furthermore, we discuss recent advances in the field of HSC biology and transplantation made in preclinical models of zebrafish, mouse, and nonhuman primates that could inform emerging practice for clinical application.

## Graphical abstract

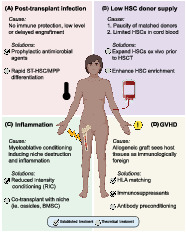

Clinical (established) and preclinical (theoretical) advances are informing treatment options for circumventing current hematopoietic stem cell transplantation (HSCT) limitations including post‐transplant infection, low HSC donor supply, inflammation, and graft‐vs‐host disease (GVHD). This review covers the advances in preclinical hematopoietic stem cell models and possible implications for improving therapeutic transplantation.

**Significance statement**

This review considers deficiencies in current hematopoietic stem cell transplantation and the fundamental insights from preclinical studies in diverse model organisms including zebrafish, mouse, and nonhuman primates.

## 1 INTRODUCTION

Hematopoiesis is one of the best understood tissue differentiation hierarchies, owed in part to the easy accessibility and processing of the tissue, and the distinctive morphologies of differentiated cell types. Classically, hematopoietic stem cells (HSCs) are defined as multipotent cells capable of both self‐renewal and differentiation that give rise to transient amplifying progenitors of varying potencies, and ultimately to the diversity of mature lymphoid, myeloid, and erythroid blood lineages. The regenerative capacity of HSCs makes them valuable in treating patients with hematopoietic disorders via HSC transplantation (HSCT) following chemotherapy or radiotherapy.

The gold‐standard functional definition of an HSC is its ability to reestablish lifelong hematopoiesis upon transplantation into a lethally irradiated recipient.^1^ This requires the graft HSCs to home to the niche, engraft and differentiate, while maintaining the “stem‐ness” properties of self‐renewal and multipotentiality. Long‐term (LT)‐HSCs are a rare, quiescent population with lifelong hematopoietic reconstitution capacity, while short‐term (ST)‐HSCs and downstream multipotent progenitor (MPP) subsets have diminished self‐renewal capacity and restricted lineage differentiation. Engraftment of both LT‐HSCs and short‐lived progenitors are required for successful HSCT to provide sustained and rapid hematopoietic reconstitution, respectively. In recent years, lineage tracing approaches have gained popularity as a way to understand clonal diversity and HSC differentiation. These approaches utilize fluorescent or genetic labels to examine relationships among HSCs and their progeny and can be used to explore HSC dynamics not only following transplantation but also by providing insight into native, unperturbed hematopoiesis.

Herein, we describe HSCT limitations and some of the preclinical approaches being investigated to overcome these roadblocks. Furthermore, we discuss the impact of niche damage and inflammation on hematopoiesis and reconstitution, and the novel methods being employed to diminish toxicity to the niche. Finally, we explore the variety of transplantation and lineage tracing approaches utilized to further our fundamental understanding of HSC biology with an eye toward improving therapeutic HSCT in patients.

## 2 HSCT IN THE CLINIC

HSCT is used to treat many malignant, congenital, and acquired diseases, and depending on the indication for transplant, it provides replacement of the hematopoietic system, a graft‐vs‐leukemia (GVL) effect, or correction of a hematopoietic defect.^2^ In high‐risk hematologic malignancies such as acute myeloid leukemia, acute lymphoblastic leukemia, or aggressive lymphomas, HSCT is utilized after chemotherapy or radiotherapy to consolidate patient remission and provide a durable cure.^3^ HSCT is curative for certain nonmalignant disorders, such as aplastic anemia, heritable hemoglobinopathies, and immunodeficiency syndromes.^4^ There are two main types of human transplantation: autologous, in which the patient's own stem cells are harvested in advance and then reinfused post‐treatment; and allogeneic, in which a separate individual donates HSCs for infusion. Determining the applicable HSCT type is based largely on the diagnosis. For example, an autologous graft is typically utilized after high‐dose chemotherapy for solid tumors, multiple myeloma, or lymphoma.^2^ The uses for allogeneic HSCT, however, are more varied. Allogeneic HSCT are used in malignant settings for hematopoietic rescue after intensive chemotherapy, providing a GVL effect that can increase the likelihood of leukemia eradication. Healthy donors can also provide a genetic correction or phenotypic enzyme replacement for hereditary conditions like storage and metabolic disorders, immunodeficiencies, and hemoglobinopathies.^2^

Donor HSCs are harvested via bone marrow aspiration or apheresis of growth factor‐mobilized HSCs in the peripheral blood. Alternatively, umbilical cord blood is collected immediately postnatally. Although cord blood units are small in volume they are rich in HSCs and immunologically naïve T‐cells, which allows greater latitude in Human Leukocyte Antigen (HLA) matching between donor and recipient.^5^ The cell source chosen depends on multiple patient‐ and diagnosis‐related factors, as well as the availability of appropriately‐matched donors.

A preconditioning regimen is given in preparation for transplantation to clear endogenous hematopoietic stem and progenitor cells (HSPCs) in the marrow niche thereby creating space for the incoming graft, to suppress the host immune system capability to reject the foreign graft cells, and to clear the disease that the transplant is intended to treat. Preconditioning processes are termed myeloablation and immunoablation, and each can be intensified or reduced based upon the needs of the patient and the underlying indication for transplant. For example, a nonmyeloablative, or reduced intensity conditioning (RIC) regimen may be used when the patient is too ill or frail to tolerate the intensity of standard myeloablative therapy.

## 3 ADDRESSING CURRENT TRANSPLANT LIMITATIONS

**FIGURE 1:**
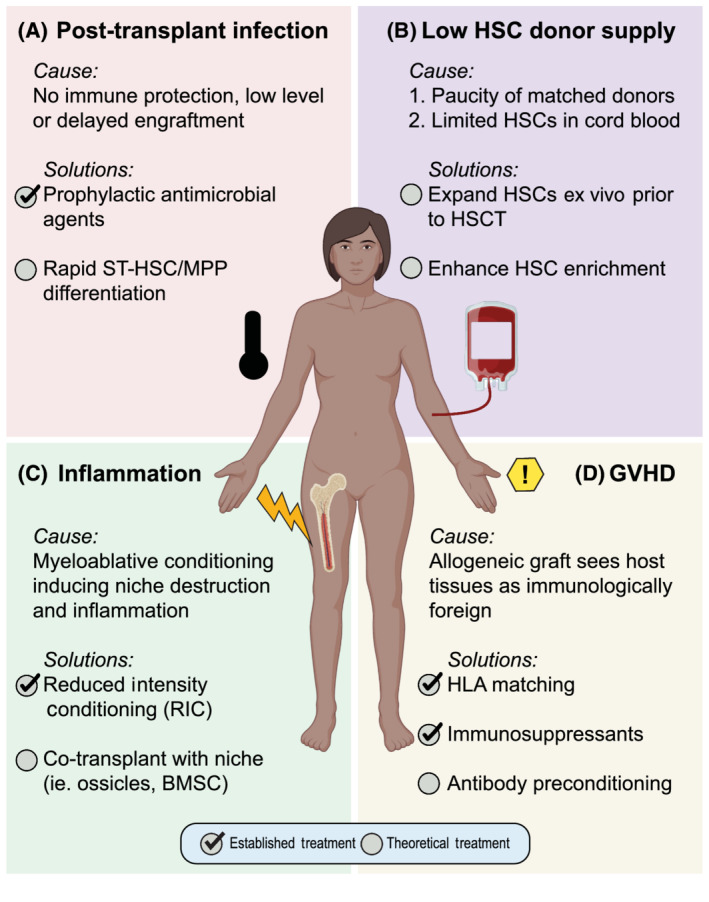
Addressing HSCT limitations. Clinical (established) and preclinical (theoretical) advances are informing treatment options for circumventing current HSCT limitation including, A, post‐transplant infection, B, low HSC donor supply, C, inflammation, and D, graft‐vs‐host disease (GVHD). Check mark indicates an established treatment in humans, while open circles signify ongoing preclinical research. BMSC, bone marrow stromal cell; HLA, human leukocyte antigen; HSC, hematopoietic stem cell; HSCT, hematopoietic stem cell transplantation; MPP, multipotent progenitors; ST‐HSC, short‐term HSC

Challenges remain in the HSCT field despite immense advances in conditioning regimens, immune matching, graft manipulations, and supportive care. A critical issue post‐transplant is low‐level or delayed engraftment (Figure 1A). This greatly increases the risk of infection, which remains one of the highest causes of post‐HSCT mortality, particularly during the postconditioning phase of neutropenia.^6^ Prophylactic antimicrobial agents are commonly administered to high‐risk HSCT patients to mitigate the risk of viral, bacterial, or fungal infection; however, such treatments carry risk of their own adverse effects including drug‐related complications, antimicrobial resistance and host microbiome disruption.^6^ Preclinical investigations aimed at understanding the processes of stem cell homing and engraftment are the first step toward improving the speed of engraftment and immune reconstitution, which would lessen the post‐engraftment infection risk enormously.

The effect of cell dose on both engraftment speed and risk of graft rejection has long been known.^7^ A major challenge in human HSCT is obtaining appropriate and sufficient donor cells for robust hematopoietic repopulation. This stems from two issues: a paucity of matched donors, and limited HSPCs obtained from cord blood units (Figure 1B). Utilizing a donor HLA matched as fully as possible to the recipient is necessary to avoid the morbidity and mortality of graft‐vs‐host disease (GVHD), but for many patients who require HSCT, especially patients of racial and ethnic minorities, fully matched donors are unavailable in donor registries.^8^ The advantage of cord blood is that the naïve T‐cells contained within the graft allow for more HLA mismatching between donor and recipient without increasing the risk of GVHD. However, the number of HSCs obtained within a single cord blood unit are often insufficient to repopulate hematopoiesis in a large adult host,^9^ and even when successful, delayed hematopoietic reconstitution leaves the recipient open to increased risk of infection and bleeding. As recently reviewed in Sica et al,^10^ multiple ex vivo strategies are being developed for graft manipulation and expansion while still maintaining HSC self‐renewal and differentiation capacities, including co‐culture with cytokines and growth factors.^11^

In preclinical studies, humanized ossicles have been engineered to facilitate targeted investigation of HSC interactions with the niche in healthy and malignant hematopoietic settings.^12^ Humanized ossicles are bone marrow‐like organoids generated using primary human mesenchymal stem cells (MSCs) that are embedded in scaffolds and implanted into mice. By recreating the HSC niche or microenvironment, researchers aim to not only interrogate niche‐HSC interactions, but also to promote in vivo expansion of HSCs for transplantation. The use of stromal cells and/or animal‐derived serum and growth factors in preclinical expansion models are inappropriate for clinical applications. To address this, protocols advancing cell purification and ex vivo graft manipulations that enhance HSC replicative potential are in development. For example, culture in defined, serum‐free media with a polyvinyl alcohol (PVA) substrate has enabled massive expansion of both mouse and human umbilical cord HSPCs, while maintaining multilineage engraftment capacity upon serial transplantation in mice.^13^ In addition, co‐culture with endothelial cells derived from primary bone marrow or human umbilical vein endothelial cells (HUVECs) mimics the HSC microenvironment and has been shown to support ex vivo expansion of mouse, nonhuman primate and human HSPCs while maintaining multilineage engraftment and serial repopulation capacity.^10^, ^14^, ^15^, ^16^, ^17^, ^18^ In vivo approaches are also being pursued to improve engraftment or repopulation capacity of available HSCs, for example Tumor Necrosis Factor (TNF)‐α inhibition post‐transplant, which has been shown in murine models to improve engraftment.^19^ However, further optimization is required before any of these techniques can be utilized clinically. Overall, an array of methods to culture and/or support HSC expansion and repopulation capacity are under development in preclinical and clinical models, and translation of these findings could overcome the limitation of low HSC numbers in cord blood grafts.

The toxicity of HSCT conditioning regimens also requires improvement. Preconditioning with myeloablative radiation to clear the bone marrow for HSCT significantly disrupts the bone marrow microenvironment, damaging the endothelium and triggering inflammatory signaling cascades (Figure 1C). HSCs reside in the bone marrow within a perivascular niche composed of a complex milieu of cells, extracellular matrix, substrate tension, and soluble factors.^20^, ^21^ It is well‐established that bone marrow vasculature functions not only as the circulation conduit, but also actively contributes to niche signaling to regulate HSC self‐renewal, differentiation, and regeneration.^22^ Sublethal myeloablative irradiation depletes vascular and perivascular cells in the bone marrow via p53 pathway activation.^23^ In turn, the stress on bone marrow endothelial cells causes HSC depletion and egress from the bone marrow to the periphery.^23^ Myelosuppressive drugs or lethal irradiation in mice induce release of inflammatory cytokines and significant bone loss due to increased osteoclast activity.^24^ Consistent with these findings, elevated levels of bone turnover markers persist for 12 months after therapy in human patients following HSCT.^24^ Combined, these studies demonstrate that the architecture and signaling of the HSC microenvironment are disrupted following myeloablation, which impairs HSC engraftment and function.

Preclinical efforts to decrease the deleterious effects of myeloablative conditioning on the marrow niche include investigations into the potential utility of bone marrow stromal cells (BMSCs). In a murine model, freshly isolated primary CD73^+^CD105^−^SCA1^+^ BMSCs were co‐transplanted with the stem cell graft and were shown not only to reconstitute stromal function but also to improve HSC reconstitution and abundance after HSCT.^25^ As an alternative to the toxic myeloablative radiation, the explosion of RIC regimens and indications where they may be used with success has revolutionized the transplant field, opening up this therapy to patients who were previously considered too elderly, too sick, or with pre‐existing organ damage too severe. Unfortunately, RIC is not currently applicable for all patients or conditions, and there is a risk balance when choosing RIC over conventional myeloablation. With improved understanding of the stem cell niche, and more targeted, less‐toxic conditioning therapies, HSCT may become safer and more widely available.

One unique inflammatory consequence encountered in allogeneic HSCT is GVHD, in which the immune system of the graft sees host tissues as immunologically foreign and mounts an immune attack (Figure 1D). The inflammatory pathway activation in acute and chronic GVHD lead to poor outcomes for the recipient.^26^ Acute GVHD pathogenesis involves the activation of antigen presenting cells and donor T cells by foreign host cell antigens, and results in host tissue injuries.^26^, ^27^ In humans, acute GVHD manifests within the first 100 days post‐transplant, and varies in intensity.^28^ In chronic GVHD, inflammation is mediated by alloreactive CD4^+^ T cells, B‐cell activation, and generalized fibrosis/sclerosis perpetrated by macrophages.^26^ Chronic GVHD may result as progression from acute GVHD or arise de novo.^28^ Common pro‐inflammatory cytokines (ie, TNF‐α, interferons (IFNs), interleukin (IL)‐6, and IL‐1) that are produced in transplantation and contribute to GVHD are known to influence HSC engraftment and function.^29^, ^30^, ^31^

Despite advances in understanding the pathophysiological underpinnings of GVHD, the clinical reality and resultant mortality of transplant patients indicate that there is more work to be done. GVHD is a morbid and sometimes fatal condition, necessitating significant prophylactic immune suppression treatments post‐transplant until immune tolerance develops.^32^ While profoundly immunocompromised, HSCT recipients are at risk for potentially fatal opportunistic infections and viral reactivations. To limit the risk and extent of GVHD, great attention is paid to ensuring that donors and recipients are as genetically similar as possible, using the HLA matching system.^2^, ^33^ Through serologic or genetic sequencing methods, donor‐recipient pairs are typically compared at 10 HLA alleles, with higher degrees of matching conferring a higher chance of successful engraftment and lower risk of GVHD.^33^ Related donors are favored for their significant genetic similarity even outside the 10 HLA alleles.^33^ GVHD risk can be diminished by transplanting purified stem cell populations, although the risk of graft failure and rejection increases with such approaches. Mega doses of enriched CD34^+^ cells have been attempted to overcome the graft rejection barrier, although the optimal conditions for this approach are still being investigated in clinical trials.^34^, ^35^

Antibody cocktail conditioning regimens to deplete host HSCs from their niche and suppress host immune responses are under development to increase the engraftment success of purified stem cell populations in mice, including antibodies targeting CD117/c‐Kit, CD47, NK‐cells, and T‐cells.^36^, ^37^ Translating these antibody‐based conditioning regimens into the clinic would exploit the GVHD‐protective benefit of purified HSC grafts while also improving the engraftment success of this approach. Already, an anti‐cKit antibody‐based HSCT approach is being evaluated in a clinical trial for pediatric patients with severe combined immunodeficiency.^38^ The ability to potentially replace classical myeloablative conditioning for certain indications for HSCT, such as hematopoietic replacement therapy in nonmalignant diseases, with these antibody‐based regimens would be advantageous by drastically reducing the hyper‐inflammation caused by current myeloablative conditioning approaches.

## 4 FUNDAMENTAL INSIGHTS INTO HSPC BIOLOGY AND FUNCTION FROM MANIPULATIONS OF PRECLINICAL TRANSPLANTATION AND LINEAGE TRACING

Studies in preclinical animal models have laid the foundation for much of our understanding of HSCs and their utility in transplantation. Scientists employ variations of HSCT, such as limiting dilution, single cell transplantation, and serial repopulation, to elucidate distinct characteristics of HSCs. Limiting dilution analysis allows quantification of HSC frequency in the population, in which a dilution series of donor cells are competitively transplanted into a recipient cohort and HSC frequency in purified hematopoietic populations can be statistically determined.^39^ Potential clinical applications that arise from limiting dilution experiments include the identification of pathways that influence HSC self‐renewal such as canonical Wnt signaling,^40^ and the discovery of agents such as prostaglandin^41^ and estradiol^42^ that increase HSC engraftment frequency. If transferable to the clinic, these discoveries may enable human HSCT using ever smaller doses of stem cells.

The inventions of flow cytometry and monoclonal antibody manufacturing revolutionized the prospective identification of mammalian HSCs. Coupling these technologies with single cell transplantation has led to increasingly refined immunophenotypic definitions of murine and human HSPC subpopulations. Current HSC purification approaches in murine models utilize lineage^−^Sca1^+^c‐Kit^+^ (LSK) and Signaling Lymphocyte Activation Molecule (SLAM; CD48/CD150) markers in combination with single cell transplantation functional analysis.^43^ Four MPP subsets have been identified and extensively studied, revealing distinct immunophenotypes, lineage bias, and cell cycle status.^44^, ^45^ In addition, serial transplantation is useful for defining the self‐renewal capacity of HSCs. Studies in animal models have shown progressively reduced output with repeated HSC isolation and transplantation, suggesting a state of HSC exhaustion.^46^ This assay has facilitated identification of critical regulators of HSC self‐renewal capacity such as reactive oxygen species (ROS)^47^ and epigenetic regulators *Ezh2* ^48^ and DNMT3A.^47^, ^49^

The impact of post‐transplant inflammation is not restricted to the niche. Inflammatory signaling also has important consequences on donor‐derived HSC function, aging, and lineage output. In murine HSCT, inflammatory signaling increases apoptosis of differentiated cells and HSCs, and alters HSC differentiation.^31^, ^50^ Specific to the transplantation procedure, a recent study demonstrated that irradiation damage of murine bone marrow induced ROS accumulation and elevated TNFα levels in transplanted HSCs.^29^ This finding is important because TNFα receptors negatively regulate HSC terminal differentiation and expansion.^51^ Moreover, HSCT upregulates IFNγ and IL‐1, which affects HSC subset differentiation, skewing toward myeloid lineages.^45^, ^50^, ^52^ Overall, transplantation‐related inflammation and altered niche impairs HSC function and engraftment, highlighting the need to understand the HSC‐niche interplay within the healthy, intact setting in order to improve transplant outcome.

**FIGURE 2:**
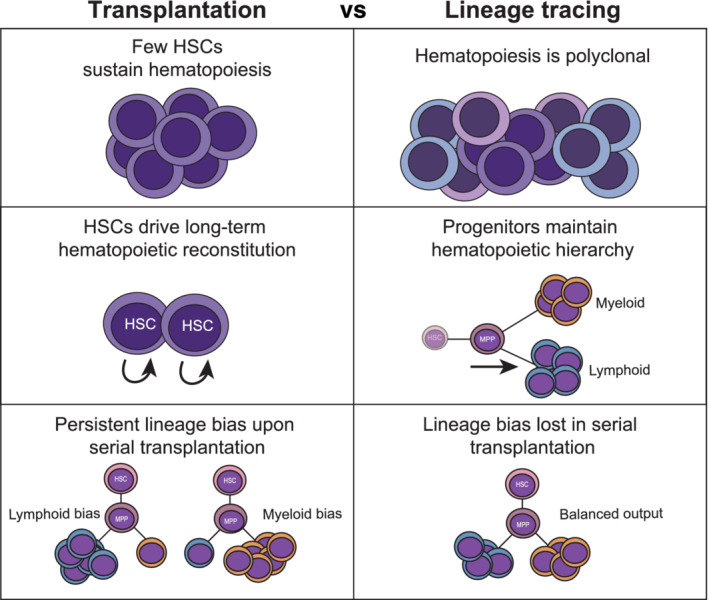
Lessons about HSC biology from transplantation and lineage tracing. Differences in how hematopoiesis is sustained were revealed following transplantation (left) or using lineage tracing methods (right). BMSC, bone marrow stromal cell; GVHD, graft‐vs‐host disease; HSC, hematopoietic stem cell; MPP, multipotent progenitor

It is critical to have a comprehensive understanding of normal HSC biology to optimally utilize them in a therapeutic HSCT setting. Building on insights about HSCs from HSCT studies, in vivo genetic lineage tracing systems have been employed in murine, zebrafish, and nonhuman primate models to genetically or fluorescently label HSC clones and their progeny allowing reconstruction of lineage fate maps.^53^, ^54^ The earliest clonal marking studies assessed HSC differentiation via the transplantation of retrovirally‐transduced HSCs.^55^, ^56^ Through this work, they not only found that a small number of HSC clones could reconstitute and maintain hematopoiesis in recipients, but they also provided the foundation for modern gene therapy. Reports tracking the reconstitution of transplanted fluorescently‐barcoded cells support the finding that few HSC clones are needed to reconstitute irradiated organisms.^57^, ^58^ However, steady‐state lineage tracing analysis demonstrates that many different HSC clones maintain homeostatic blood production, suggesting HSC contribution is polyclonal or heterogenous at baseline.^57^, ^58^ Additionally, lineage tracing has supplemented our understanding of lineage‐biased HSC subsets which are primed to produce certain cell types over others. Lineage bias identified in HSCT is retained upon serial transplantation,^59^, ^60^ but megakaryocyte/platelet‐biased HSC subsets in a steady‐state context do not maintain their primed state and instead revert to multipotency upon HSCT.^61^ Lastly, in the native, unperturbed environment unaffected by myeloablation and transplantation, it is downstream progenitors, not HSCs, that drive the production and replenishment of most myeloid and lymphoid committed cell types, as determined using lineage tracing strategies in combination with singlecell sequencing.^61^, ^62^, ^63^ This finding contrasts with clonal development studies in a transplantation setting, which place HSCs as the main drivers of differentiation.^64^, ^65^, ^66^ Therefore, HSCs drive multilineage differentiation under stress conditions (ie, transplantation), but under steady‐state conditions, their progenitors are largely responsible for maintaining hematopoiesis (Figure 2). The lessons learned from steady‐state in vivo analyses will elucidate the optimal conditions for HSC function, providing key information regarding HSC behavior that could be used to improve HSCT. The importance of multilineage progenitors in maintaining lifelong hematopoiesis has been highlighted from the numerous lineage tracing studies, indicating the need to further explore these cells for clinical usage.

While hematopoiesis is highly conserved in vertebrate and mammalian models, a challenge remains to translate the significance of mouse and zebrafish findings into human HSCT practice. Nonhuman primate transplantation studies offer a valuable preclinical bridge for this, due to their closer phylogenetic proximity to humans compared with mice and zebrafish.^67^, ^68^ Early work tracking the cell cycle status of baboon HSPCs indicated these cells are extremely quiescent, consistent with the murine lineage tracing studies demonstrating minimal activity of HSCs during steady‐state hematopoiesis.^69^ Using rhesus macaque autologous transplantation with lentiviral cellular barcoding, the Dunbar lab demonstrated that contributions from short‐term HSCs and uni‐lineage engrafting progenitors disappeared by 2‐3 months post‐transplantation, while long‐term HSC contributions persisted with similar clonal dynamics beyond 3 months.^67^ In contrast to the low number of HSCs contributing in murine transplantation, long‐term HSC contribution in macaques was clonally diverse even at 38 months post‐transplantation with no single clone predominating.^68^ Interestingly, reports identified that some HSCs or multipotent progenitors have a strong skewing to produce natural killer cell lineage (CD16^+^CD55^−^), lacking clonal overlap with T, B or myeloid cells.^67^ These lineage tracing approaches in nonhuman primates further add to the information garnered from mice and zebrafish investigations and provide invaluable insight that could further guide human HSCT.

Although more limited, lineage tracing is also possible in humans following transplantation and in steady‐state hematopoiesis. For example, clonal tracking in gene therapy patients is one way to ethically conduct lineage tracing studies into human hematopoiesis.^70^ Integration sites from the ex vivo transduction of HSCs with a therapeutic vector allows for direct lineage tracing of transplanted cells in these patients. Estimates of the number of active, repopulating bone marrow CD34^+^ HSCs are around 2,000‐50 ,000 cells, which positively correlated with the number of HSCs originally infused, and myeloid‐, lymphoid, and balanced HSCs subsets were among these active HSCs. More recently, innovative deepsequencing approaches have opened the door to study native human hematopoiesis. The identification of endogenous somatic mutations in bulk blood cell populations in healthy donors using genomic sequencing permitted the tracking of hematopoietic clones over time without the need for genetic manipulation or transplantation.^71^ From this study, it was computationally determined that HSC self‐renewal occurs in the range of 2‐20 months and approximately 50,000‐200,000 total HSCs actively contribute to hematopoiesis in adult humans. Comparing HSCs by their somatic DNA changes revealed that human hematopoiesis is polyclonal and maintained by cells exhibiting multipotency.^72^ As seen in murine, zebrafish, and nonhuman primates models, heterogenous HSC contribution to human hematopoiesis is supported by the lineage tracing studies with evidence of HSC lineage priming toward megakaryocytes^72^ or other mature blood cell types.^71^ Deep targeted sequencing for somatic mutations is both costly and highly error‐prone, but recent studies detecting mtDNA mutations with bulk Assay for Transposase‐Accessible Chromatin using sequencing (ATAC‐seq) or singlecell RNA‐sequencing are establishing more cost‐effective lineage tracing approaches that have similar utility to exogenous lentiviral barcoding.^73^, ^74^ Lineage tracing with mtDNA has provided additional evidence for the heterogeneity of the “preleukemic” HSC population that evolve into acute myeloid leukemia.^74^ Genomic and mitochondrial mutations are thus providing new ways to investigate human HSC properties in situ and add to our existing knowledge of human hematopoiesis.

## 5 CONCLUSIONS

Over recent decades, the remarkable improvements in survival rates and other outcomes after HSCT are owed in large part to vast improvements in our understanding of both transplantation and hematopoiesis, as well as enhanced supportive care. Despite this, our understanding of the underlying biologic processes in HSCT is still limited. Transplantation models and lineage tracing provide powerful tools to better study and understand perturbed and native hematopoietic environments, including lineage fate choices. Employing both transplantation and lineage tracing is bridging the gap between model organism and human hematopoietic studies, facilitating refinement of the hematopoietic hierarchy in normal and diseased states. Preclinical models, and the insights gained from HSCT manipulations and lineage tracing experiments, have enabled unparalleled exploration of the hematopoietic system and its ability to engraft a new host. The translation of these insights to the clinic will enable patients requiring transplant to potentially experience less toxicity, diminished inflammation, and improved graft HSC function, all with a smaller input cell dose. These advances would not only make HSCT safer and more accessible for wider ranges of currently ineligible patients, but they would also drastically improve morbidity and mortality.

## CONFLICT OF INTEREST

The authors declared no potential conflicts of interest.

## AUTHOR CONTRIBUTIONS

E.F., B.A.U., M.F.N., K.S.P.: conception and design, analysis and interpretation, manuscript writing; T.V.B.: conception and design, manuscript writing, final approval of manuscript.

## Data Availability

Data sharing is not applicable to this article as no new data were created or analyzed in this study.
